# Auriculotherapy to reduce anxiety and pain in nursing professionals: a randomized clinical trial

**DOI:** 10.1590/1518-8345.1761.2843

**Published:** 2017-04-06

**Authors:** Leonice Fumiko Sato Kurebayashi, Ruth Natalia Teresa Turrini, Talita Pavarini Borges de Souza, Carolina Felicio Marques, Renata Tavares Franco Rodrigues, Karen Charlesworth

**Affiliations:** 1 Post-doctoral fellow, Escola de Enfermagem, Universidade de São Paulo, São Paulo, SP, Brazil. Scholarship holder at Programa Nacional Pós Doutorado da Coordenação de Aperfeiçoamento de Pessoal de Nível Superior (CAPES).; 2 PhD, Full Professor, Escola de Enfermagem, Universidade de São Paulo, São Paulo, SP, Brazil.; 3 Doctoral student, Escola de Enfermagem, Universidade de São Paulo, São Paulo, SP, Brazil. Professor, Escola de Enfermagem São Joaquim, Hospital Beneficência Portuguesa, São Paulo, SP, Brazil.; 4 Professor, Escola de Enfermagem São Joaquim, Hospital Beneficência Portuguesa, São Paulo, SP, Brazil.; 5 MSc, Professor, Escola de Enfermagem São Joaquim, Hospital Beneficência Portuguesa, São Paulo, SP, Brazil.; 6 Specialist in em Acupuncture, Researcher, Northern College of Acupuncture, York, North Yorkshire , United Kingdom.

**Keywords:** Auriculotherapy, Anxiety, Pain, Quality of Life, Protocol

## Abstract

**Objectives:**

to evaluate the effectiveness of the auricular protocol (APPA) in reducing pain and anxiety and improving the quality of life of the nursing staff of a hospital.

**Method:**

randomized clinical trial with an initial sample of 180 professionals divided into 4 groups Control (G1), Seed (G2), Needle (G3) and Tape (G4). The evaluation instruments were the State-Trait Anxiety Inventory, Pain Visual Analog Scale and Quality of Life instrument, applied at the start and after five and 10 sessions (five weeks). Descriptive statistics, analysis of variance (ANOVA) and Cohen's d Index were used in the analysis.

**Results:**

there was a statistical difference (p < 0.05) for anxiety according to the repeated measures ANOVA, with better results for the G3 in the final assessment (Cohen's d index 1.08/17% reduction). There was a reduction of pain of 36% in G3 and 24% in G2 and a 13% increase in the mental aspect of quality of life for the G3, although without statistical significance.

**Conclusion:**

the APPA protocol reduced the anxiety levels of nursing staff after 10 sessions. Further studies are, however, suggested with new populations and in different contexts so that the results can be confirmed. RBR-5pc43m.

## Introduction

Mental health is one of the great challenges of the twenty-first century. Considering this, a study revealed that 30% of the population of the metropolitan area of São Paulo suffered from some kind of mental disorder. Among the most common problems identified in the study were anxiety, behavioral changes and substance abuse. Anxiety was present in 20% of the respondents. The study confirmed a greater prevalence of mental disorders in adults in São Paulo than those of similar study conduct in other area of the world^1^.

Although the individual clinical and sociocultural factors need to be considered, contextual factors inevitably have a heavy impact on the development of mental problems. Among these, the economic crisis and rising unemployment levels can certainly contribute to a sense of general and collective insecurity^2^. Brazil is experiencing a chaotic historical moment regarding political, economic, ethical and social aspects, with a tragic legacy of a driving motion of the economy that has acquired an astonishing rhythm of unbalance and overload over the past four years. The result of government intervention in the economy associated with corruption has caused increased inflation, credit restriction, closure of businesses and rising unemployment, coupled with a lack of faith of the population in relation to the government^3^.

The city of São Paulo had a population of 11,581,798 inhabitants in 2015 and is considered the largest city in the country and in South America^4^. In addition to the current economic and political developments, the process of rapid expansion and urbanization is an aggravating factor for the welfare of the residents, contributing to increased social insecurity, creating urban environmental problems and irregular settlements and prejudicing the quality of life^5^. This climate of dissatisfaction and personal and collective insecurity is the background of this study, which was carried out with the nursing staff of a large general hospital in São Paulo, with an average of 3,500 Nursing professionals, many of whom lived in neighborhoods a long distance from the workplace.

Nursing workers represent one of the groups of professionals most susceptible to present health problems at work, as they perform complex tasks involving a high physical and mental workload^6^. Other factors potentiate damage to their physical and mental integrity, causing pain and anxiety, such as: lack of personal protective equipment, dissatisfaction related to working conditions, low wages, difficult interpersonal relationships, lack of trust between professionals and management, and physical and emotional exhaustion due to caring for patients with pain and suffering. All this generates increased rates of absenteeism^7^.

In anticipation of proposing a preventive treatment to reduce levels of anxiety and pain in the nursing staff, the use of auriculotherapy has been proposed as a complementary and preventive practice. This proposal sought to test a protocol organized by researchers at the Northern College of Acupuncture, UK, coordinated by Hugh Mackpearson, professor and researcher of the University of York, for the formulation of a protocol for pain and anxiety (The Auricular Protocol for Pain & Anxiety - APPA), created by Karen Charlesworth. Having participated as a specialist in auricular acupuncture for the creation of the protocol with 30 other experts from other countries, in the period from June to July 2014, the idea emerged of holding the first protocol trial in a hospital in Brazil, with a Nursing team. The protocol was previously created for people living in situations of danger, conflict, disaster and poverty^8^.

While it was recognized that the scope of auricular technique can be greater when performed in an individualized way that does not follow a protocol and by professionals with theoretical and practical knowledge of diagnoses^9^, the possibility of using a protocol assists in the dissemination and popularization of the art, with the aim of benefiting more people. It should also be noted that auriculotherapy has important advantages, being easily administered, very rapid, relatively inexpensive, achievable with non-invasive materials and presenting minimal adverse side effects^10^. The aim of the study was to evaluate the effectiveness of the auricular protocol for pain and anxiety (APPA) and improving the quality of life of nursing staff of a hospital.

## Method

This was a parallel-group randomized controlled trial conducted in the Beneficência Portuguese Hospital of São Paulo, from June 2015 to February 2016. A total of 193 nursing staff employees were initially contacted and 180 people who presented high or moderate levels of anxiety, according to the State-Trait Anxiety Inventory (over 33 points), were invited to participate. Pregnant women, subjects who would go on vacation and those taking sick leave during the survey period were excluded, as were those that started using allopathic medication for anxiety or antidepressants, those that initiated other energy therapies during the study period and those that were allergic to metal or adhesive tape. However, those that were undergoing psychological therapy at this moment were not excluded, with the proviso that the treatment continued normally.

**Figure 1:**
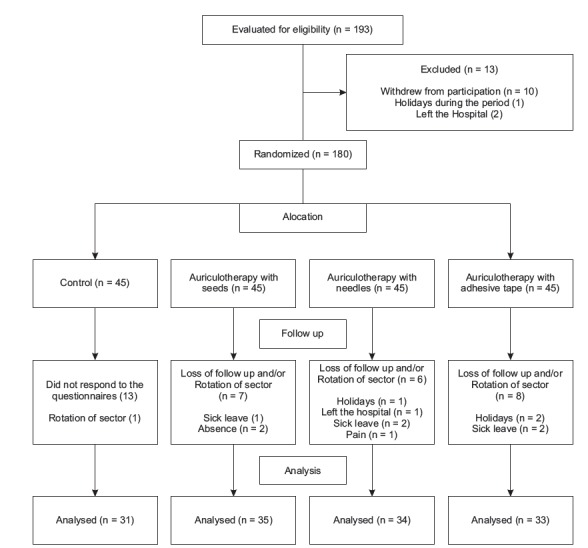
Flowchart of participants involved in the study. São Paulo, SP, Brazil, 2016

The sample size of 120 individuals was originally proposed for a test power of 80% and a 95% confidence interval. The eligible population obtained was 180 subjects and 133 completed the study (Figure 1), with a loss of 47 (26.11%) individuals.

A total of 22 people missed at least two sequential sessions in a week due to sector rotation or sequential days off; 15 did not attend the second evaluation or did not turn up to respond to the questionnaires (control); five went on medical leave during the study period, three took holidays, one person stopped working for the hospital and one gave up due to feeling pain at the zero point, which is a point located in the center of the ear between the two shells, about the root of the helix.

**Figure 2:**
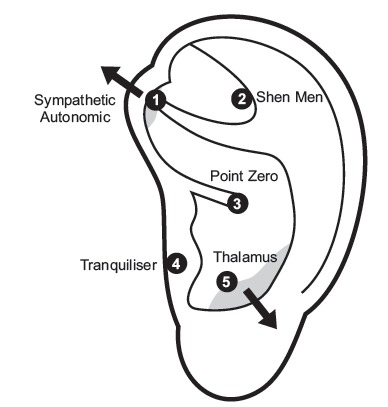
Auricular Protocol for Pain & Anxiety - APPA

Source: http://www.appa-acupuncture.org/

For the allocation of the individuals, a random division was made into four groups, using the Research Randomizer program (available at http://www.randomizer.org/form.htm): Control - G1 (without intervention) Auriculotherapy with seeds - G2, Auriculotherapy with semi-permanent needles - G3, and Auriculotherapy with adhesive tape - G4 (placebo). The treatment consisted of ten twice weekly sessions for five weeks, which were performed in the sector where the professional worked, during the work time, with duration of 5 to 10 minutes. The protocol used was the beta version of the Auricular Protocol for Pain & Anxiety - APPA: using the Shenmen, tranquilizer, thalamus, autonomic system or sympathetic points and the zero point, as shown in Figure 2. The protocol was applied unilaterally in each session.

After the location of the reactive points with a manual probe, the cleaning of the ear with cotton and 70% ethyl alcohol was performed, followed by the application of the different materials: seeds, semi-permanent needles and non-allergenic adhesive tape at the same points. The needles remained in place for two days, if there was no discomfort. For the seeds, the subjects were instructed to stimulate them 15 times, at least three times a day.

The therapists (n = 5) were health professionals, one psychologist and four nurses, trained in auriculotherapy by the Institute of Integrated and Eastern Therapy. All participants completed a biosociodemographic questionnaire and the instruments: Spielberger's State-Trait Anxiety Inventory (STAI)^11^, Visual analogue scale (VAS) of pain and SF-12v2 Quality of life^12^, applied at the beginning, after five sessions and at the end of ten sessions.

The study fulfilled the requirements of resolution 466/12 of the National Council of Health Ethics of Brazil for research involving human subjects, and all the subjects signed the consent form. Participants in the Control and Adhesive Tape groups were offered the opportunity to receive auriculotherapy after completion of the study. The research project was approved by the Research Ethics Committee of the School of Nursing of the University of São Paulo (No. 1.105.429), by the Committee of the Hospital and was registered with the Brazilian Registry of Clinical Trials (No. RBR-5pc43m).

For the descriptive analysis of the data, measures of central tendency and absolute and relative frequencies were used, with the repeated measures ANOVA used to compare the groups. Also Cohen's d index was used to measure the effect size and the percentage of change in the three moments (before, after 5 and after 10 sessions).

## Results

The mean age of the participants was 35.7 years (± 8.4), 84.2% (n = 112) were female, 43.6% (n = 8) single, 48.1% (n = 64 ) married, 67.7% (n = 90) with mid or technical level education, 76% (n = 101) had no history of previous illness, 94.7% (n = 126) did not use tranquilizers, and only 1.5% (n = 2) were undergoing psychotherapeutic treatment. The sample was comprised of 36% (n = 48) nursing assistants, 42.1% (n = 56) nursing technicians and 21.8% (n = 29) nurses; 32.3% (n = 43) worked on the morning shift and 63.2% (n = 84) on the afternoon; 33.8% (n = 45) were from the Hemodynamic and Hemodialysis sectors, 29.3% (n = 39) from the Intensive Care Unit and Emergency Room and 17.3% (n = 23) from the Imaging sectors (X-ray, endoscopy, volume, resonance, nuclear medicine). At baseline, they presented moderate levels of state anxiety 49.7 (± 9.0), trait anxiety 45.7 (± 9.5), mean pain of 4.6 (± 3.0), mean of the physical domain of the SF-12v2 of 46.1 (± 7.9) and mean of the mental domain of the SF-12v2 of 43.9 (± 10.6). Of the total of 180 subjects (100%) only 33 (18.3%) reported comorbidities. A total of 40 diseases (100%) were found: Circulatory and vascular system: arrhythmia (n = 2 or 5%), hypertension (n = 5 or 12.5%), varices (n = 1 or 4%); Respiratory system: asthma (n = 5 or 12.5%), sinusitis (n=3 or 7.5%), bronchitis (n = 2 or 5%); Musculoskeletal system: osteoarthritis of knee (n = 2 or 5%), bone spur (n = =1 or 4%), plantar fasciitis (n = 1 or 4%), fibromyalgia (n = 1 or 4%), herniated disk (n = 3 or 7.5%), thumb arthritis (n = 1 or 4%) degeneration of the spine (n = 1 or 4%), chondromalacia patellae (n = 1 or 4%); Endocrine system: *diabetes mellitus* (n = 2 or 5%), hyperthyroidism (n = 1 or 4%), hypothyroidism (n = 1 or 4%); Digestive System: gastritis (n = 1 or 4%), irritable bowel syndrome (n = 1 or 4%) and others: hearing loss (n = 1 or 4%), migraine (n = 3 or 7.5%), myoma (n = 1 to 4%).

There was homogeneity in the distribution between the groups regarding all sociodemographic variables and levels of anxiety, pain and quality of life (p > 0.05), according to Fisher's exact test and the ANOVA. In the analysis of moments 3 and 1, according to the repeated measures ANOVA, there was no statistical difference (p = 0.028). The treatment with needles reduced anxiety levels, with a Cohen's d index of 1.08 (Large effect) and 17% reduction (Moderate decrease) (Table 1).

**Table 1:** Means and standard deviation of STAI-state levels, pain visual analogue scale, physical (SF12 F) and mental (SF12 M) domains of the quality of life scale, in the four groups and at the three moments. São Paulo, SP, Brazil, 2016

Group		n	Moment 1	Moment 2	Moment 3	Moment 3-1	
			Mean (SD)	Mean (SD)	Mean (SD)	Cohen's *d*	%
STAI State							
	Control	31	48.0 (9.3)	47.9 (10.2)	46.7 (10.4)	0.14	-3
	Seed	35	49.3 (7.9)	43.9 (9.5)	42.8 (10.5)	0.71	-13
	Needle	34	51.6 (9.8)	44.3 (8.3)	42.9 (6.3)	1.08	-17
	Adhesive tape	33	49.5 (8.7)	46.1 (11.2)	44.1 (8.9)	0.63	-11
VAS							
	Control	31	4.7 (2.9)	4.7 (2.6)	4.51 (2.7)	0.09	-5
	Seed	35	4.3 (2.9)	3.7 (2.3)	3.3 (2.6)	0.39	-24
	Needle	34	4.6 (3.3)	3.4 (2.5)	2.9 (2.6)	0.56	-36
	Adhesive tape	33	4.6 (2.9)	4.5 (3.2)	4.4 (3.3)	0.08	-5
SF12 physical							
	Control	31	46.3 (8.7)	44.3 (8.8)	44.5 (8.8)	0.21	-4
	Seed	35	45.3 (8.3)	45.3 (10.4)	46.8 (7.5)	0.18	3
	Needle	34	45.6 (7.8)	48.4 (7.2)	47.7 (6.5)	0.3	5
	Adhesive tape	33	47.1 (7.04)	45.5 (9.9)	47.8 (7.2)	0.1	1
	SF12 mental						
	Control	31	45.7 (10)	45.1 (10.7)	47.7 (8.3)	0.22	4
	Seed	35	44.3 (10.7)	46.8 (8.8)	47.1 (9.5)	0.28	6
	Needle	34	43.5 (9.9)	44.8 (10.7)	49 (7.3)	0.64	13
	Adhesive tape	33	42.2 (11.9)	45.36	44.4 (10.8)	0.19	5

**Figure 3:**
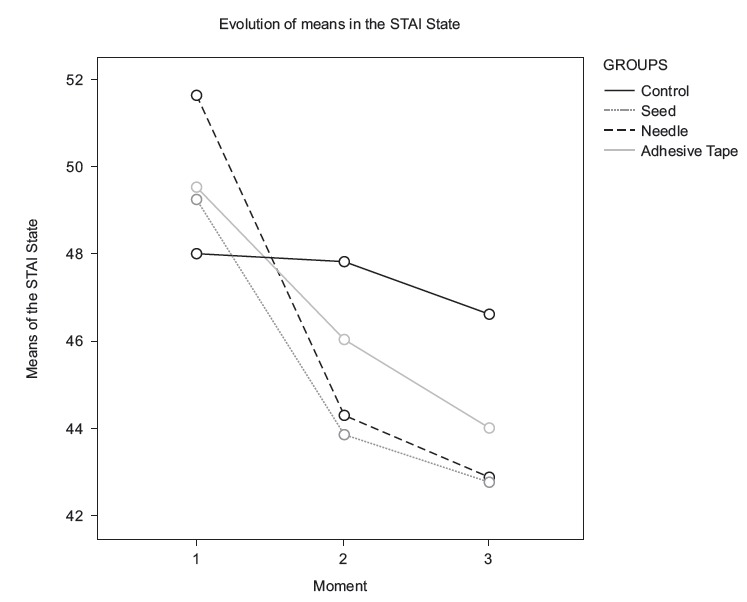
Evolution of state anxiety levels in the four groups, at the three moments. São Paulo, SP, Brazil, 2016

**Figure 4:**
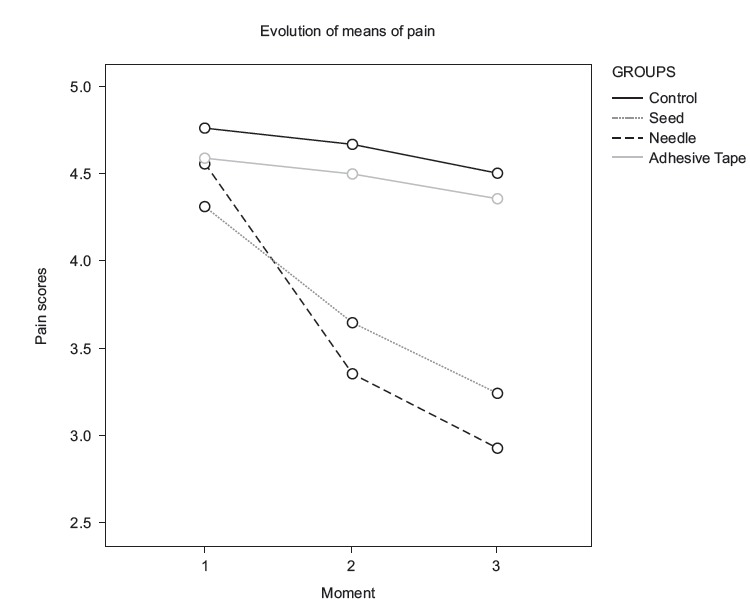
Evolution of pain levels (VAS) in the four groups, at the three moments. São Paulo, SP, Brazil, 2016

Regarding the other instruments, there was no difference between the moments in the intergroup analysis, p = 0.169 for pain, p = 0.224 for the physical domain of the SF-12v2 and p = 0.385 for the mental domain for the moments 3-1. Cohen's *d* for the mental domain was 0.64 (Moderate effect), 13% (Small increase) for the needle group. Concerning pain levels, the Cohen's d index for the needle group was 0.56 (Moderate effect) and 36% pain reduction (Large decrease). Figures 3 and 4 illustrate the evolution of the levels of anxiety and pain respectively.

Only one person withdrew from the study due to pain with the needles. Other people who felt pain continued in the trial. The adhesive tape group did not function as a placebo, as the participants were not blinded and it also did not produce any effects.

## Discussion

The best result for reducing state anxiety was produced by the auriculotherapy with needles. Similarly, in a study conducted in a university hospital, there was a greater reduction of stress by auriculotherapy with needles compared to that with seeds, using the Shenmen, Kidney and Brainstem points. Unlike seeds, needles do not need to be stimulated, however, may produce local pain and run a risk of infection. Even with lower results, there is an advantage in the use of seeds as they produce less discomfort^13^ and can be applied by people only trained for the protocols. In this regard, the first test of the APPA protocol in Nepal obtained positive results after the earthquake that devastated the country in 2015^14^.

Crisis situations compromise the mental health and the usefulness of the APPA was only observed with regards to anxiety, perhaps because the pain level was not an inclusion criterion for the present study. Each auricular protocol has a pathophysiological rational, which often aims to approximate the Western knowledge with the Eastern, and also considers the perception that the researchers have about the phenomenon of traumatic situations. In this sense, starting from a more Eastern assumption, the NADA (The National Acupuncture Detoxification Association) protocol has been developed to reduce stress and alleviate trauma in communities suffering from disasters or conflicts. It was previously developed for the well-being of communities, mental health and the control of drug abuse and smoking, among other conditions^15^. Acupuncturists Without Borders, in 2005, used the NADA protocol in the aftermath of Hurricanes Katrina and Rita and many other disasters in the USA^16^. The points common to both protocols are the Shenmen and Sympathetic or Autonomic points.

Regarding the APPA protocol, the most painful point reported was the Zero point, when using semi-permanent needles. There was no reported discomfort in relation to the other points. The Zero point was used postoperatively in another study, as a regulator of the autonomic nervous system and of parasympathetic activity, together with the Shenmen point, with positive results based on the analysis of heart rate variability^17^.

Auriculotherapy points for anxiety were evaluated in 14 articles, with the Shenmen (64.3%) and Relaxation (28.6%) points being the most used. Other points used for emotional problems were: Brainstem, Master Cerebral, Heart, *Valium* or Tranquilizer, Sympathetic and Endocrine^18^. Auriculotherapy has also been studied as a less invasive method of vagus nerve stimulation for the treatment of patients resistant to therapy, with disorders such as depression and epilepsy. A trial used stimulation of the inferior concha achieving positive results with electro-stimulation. This area shows neuroanatomical evidence of vagal afferents(19). The Shenmen, in the triangular fossa, is a region innervated by a branch of the vagus nerve, with anti-inflammatory and calming actions^20^.

The APPA produced a reduction in the level of pain of 36% with needles and 24% with seeds. Specific points for musculoskeletal or visceral pain were not used, opting for the use of general points, such as the Shenmen, Thalamus, which are indicated for pain. It is in the thalamus that painful information is located and projected to the structures of the limbic, motor and cortical systems. Thus, the painful impulse reaches the limbic system, carrying the experience as something unpleasant and emotional, resulting in the interpretation of pain^21^. Other studies with the APPA protocol have suggested the evaluation of the effects on pain and the investigation of whether more complementary points may eventually be needed. Mental and physical health are two closely intertwined and interdependent elements of life and the APPA seeks to influence both aspects. Currently, the intrinsic and strong relationship between the physical and emotional aspects of anxiety is recognized and accepted. There is a significant correlation between pain and symptoms of anxiety and depression, especially chronic pain^22^.

Considering the limitations of the study, it was not possible to ensure the proper participation of the subjects who were treated with seeds, as the seeds need to be pressed to achieve better results. As it is important for the APPA Protocol that the auriculotherapy is made with non-invasive materials, further studies with other materials, such as magnets or magnetic crystals, are recommended. The location of the points was performed manually, without the use of electronic devices or of the atrio-cardiac reflex proposed in French auriculotherapy for the location of points. The group with adhesive tape did not function as a placebo, due to failing to blind the subjects. Perhaps the auriculotherapy with seeds would achieve better results if the number of participants was greater or if a longer treatment time was used. Other points could possibly be added or changed in the APPA protocol, in order to intensify its effect in relation to pain. It is suggested that other studies consider these aspects.

## Conclusion

The APPA protocol, when applied with the nursing staff of a hospital in São Paulo, achieved significant positive differences in the reduction of anxiety, according to the repeated measures ANOVA, after 10 sessions. The group with semi-permanent needles presented a Cohen's d index of 1.08 (large effect) and 17% reduction. There was a reduction in pain levels by 34% for the needle group and 24% for the seed group and a 13% increase in the mental domain of SF12v2 quality of life measure, although there was no statistical differences in the intergroup analysis. Further studies are suggested with new populations and in different contexts and situations so that the results can be confirmed. It is also recommended that other auricular points are tested to intensify the effects in relation to pain.
